# Fish oil supplementation, physical activity and risk of incident Parkinson’s disease: results of longitudinal analysis from the UK Biobank

**DOI:** 10.3389/fnagi.2023.1304629

**Published:** 2024-01-29

**Authors:** Fabin Lin, Yisen Shi, Jiayi Zheng, Yueping Li, Xuanjie Chen, Xinyang Zou, Yi Hong, Ke Chen, Yuqi Zeng, Qinyong Ye, Xiaochun Chen, Xinyan Chen, Yingqing Wang, Guoen Cai

**Affiliations:** ^1^Department of Neurology, Institute of Clinical Neurology, Center for Cognitive Neurology, Fujian Medical University Union Hospital, Fuzhou, China; ^2^Fujian Institute of Geriatrics, Fujian Medical University Union Hospital, Fuzhou, China; ^3^Fujian Key Laboratory of Molecular Neurology, Fujian Medical University, Fuzhou, China; ^4^School of Basic Medical Science, Fujian Medical University, Fuzhou, China; ^5^Department of Neurosurgery, Fujian Medical University Union Hospital, Fuzhou, China

**Keywords:** physical activity, fish oil, Parkinson’s disease, genetic predisposition, UK Biobank

## Abstract

**Objective:**

Evidence on the individual and combined relationship of physical activity (PA) and fish oil supplement use on the incidence of Parkinson’s disease (PD) risk remains lacking.

**Materials and methods:**

This UK population-based prospective cohort study, involving 385,275 UK Biobank participants, collected PA and fish oil supplement data via touchscreen questionnaires. Using Cox proportional hazards models and restricted cubic splines to examined the associations between use of fish oil supplements, PA and PD risk.

**Results:**

During a median 12.52-year follow-up, 2,131 participants incident PD. Analysis showed that fish oil supplement users had a lower PD risk [hazard ratio (HR), 0.89; 95% confidence interval (CI), 0.82–0.98]. The adjusted HRs for the PD incidence were 0.96 (95% CI, 0.95–0.98) for total PA; 0.93 (95% CI, 0.90–0.96) for moderate PA; 0.95 (95% CI, 0.91–0.99) for vigorous PA and 0.93 (95% CI, 0.89–0.98) for walking activity. Significant interactions were found between fish oil supplement use and total PA (*P* for interaction = 0.011), moderate PA (*P* for interaction = 0.015), and walking activity (*P* for interaction = 0.029) in relation to PD incidence.

**Conclusion:**

Both fish oil supplement use and PA were associated with a reduced risk of PD, and the effect of PA in reducing the risk of PD was more pronounced when fish oil supplement was used.

## 1 Introduction

Parkinson’s disease (PD) is the second most common neurodegenerative disorder in worldwide (Dorsey et al., 2007). It can cause a wide range of motor and non-motor symptoms, including resting tremors, muscle rigidity, slow movement, postural instability, autonomic dysfunction, cognitive impairment, and mood disorders (Cerri et al., 2019; Tolosa et al., 2021). Since 1990, PD has been the fastest-growing neurological disease in terms of prevalence, disability, and mortality worldwide (GBD 2016 Parkinson's Disease Collaborators, 2018). Considering the disease burden of PD, identifying its modifiable environmental risk factors and developing preventive interventions are important public health issues (Tysnes and Storstein, 2017; Aaseth et al., 2018; Reichmann et al., 2022).

Fish oil mainly contains the long-chain omega-3 polyunsaturated fatty acids (n-3 PUFAs), especially eicosapentaenoic acid (EPA) and docosahexaenoic acid (DHA) (Liu et al., 2022). These nutrients have a wide range of biological activities related to cardiovascular health, including lowering blood pressure and triglycerides and improving endothelial vasodilator function. Several prospective observational studies have shown an association between diets rich in n-3 PUFAs and a lower risk of PD (Avallone et al., 2019). Omega-3 fatty acids are also found to be beneficial in alleviating PD symptoms in a randomized clinical trial (RCT) (Taghizadeh et al., 2017). However, in a case–control study based on Japanese population showed that consumption of n-3 PUFAs were not significantly associated with PD risk (Miyake et al., 2010). To provide further evidence, this study explored the association between fish oil supplements and PD risk based on UK Biobank.

Physical activity (PA) has been suggested as a potentially effective means to reduce PD morbidity (Ascherio and Schwarzschild, 2016; LaHue et al., 2016; Reichmann et al., 2022). Epidemiological evidence suggested that higher levels of moderate to vigorous activity associated with a lower risk of PD (Fang et al., 2018). Besides, aerobic exercise has been found to reduce motor and non-motor symptoms in PD patients in some RCTs (van der Kolk et al., 2019; Johansson et al., 2022).

Although a large number of studies have shown that PA is recommended as a robust preventive factor for the development of PD. However, evidence for the protective effect of low-intensity PA and the amount of PA at different intensities is still limited. Considering that vigorous PA may not be feasible for some specific groups, such as morbidly obese or elderly people, and prolonged exercise may not be scientific. It is important to provide information on associations between the amount of PA and the risk of PD for different types of PA. Therefore, the present study explored the association between amount of sum PA and the risk of PD and further explored the association between amount of PA and PD risk for different types of PA (vigorous, moderate, and walking).

Previous studies have not examined the effect of the interaction between fish oil supplementation use and PA on the risk of PD. Dietary intake of fish oil has multiple potential biological mechanisms associated with increased muscle protein synthesis and improved muscle mass, which is strongly associated with loss of physical function (Smith et al., 2011; Rodacki et al., 2012). Therefore, we hypothesized that the effect of PA on the onset of PD would be modified by the intake of fish oil supplementation. Specifically, combining PA with fish oil supplementation may enhance the effect on biological pathways and thus have a greater impact on physical function. In addition, considering potential sex-specific biological responses to PA (Yang et al., 2015; Da Boit et al., 2017), it would be of interest to explore sex differences in the combined effects of fish oil supplementation and PA on the risk of PD. Moreover, as evidence suggested genetic risk may modify the effect environmental factors on PD (Jacobs et al., 2020; Bloem et al., 2021), there is a need to explore whether the relationship between fish oil, PA and PD risk is altered by differential genetic risk.

The aim of this study was to investigate the independent as well as combined effects of fish oil supplementation use and PA on the risk of PD. In addition, we further explored whether there were differences in the associations of fish oil supplement use and PA with PD risk in different sex.

## 2 Methods

### 2.1 Study population

UK Biobank is a large prospective study with 502,387 participants designed to collect detailed information on a wide range of phenotypes through questionnaires, physical measurements, sample testing, accelerometry, multimodal imaging, and long-term follow-up of a range of health-related outcomes. After excluding those with baseline PD (*n* = 900), 501,487 participants remained. We further excluded individuals with missing data for age, sex, race, education, and Townsend deprivation index (TDI), smoking, drinking, and body mass index (BMI), PA, and fish oil supplements use. Ultimately, 385,275 participants were included in this study. The detailed screening process is presented in Supplementary Figure S1.

The North West Multi-Centre Research Ethics Committee approved the UK Biobank study, and all participants provided written informed consent to participate. The study protocol is publicly available on the UK Biobank website.^1^

### 2.2 Parkinson’s disease diagnosis

As recommended by the UK Biobank [Algorithmically-defined outcomes (ADOs) Version 2.0, 2022], algorithmically-defined outcomes were used to identify PD onset in the cohort’s participants. PD definition is shown in detail in Supplementary Table S1. The disease information was obtained from inpatient electronic health records and death registers, which are linked to the Hospital Episode Statistics for England, Scottish Morbidity Record, or Patient Episode Database for Wales. The censoring dates for these databases were 31 October 2022, 31 July 2021, and 28 February 2018, respectively. The follow-up time was calculated from baseline to the time of PD diagnosis, death, loss to follow-up, or censorship, whichever occurred first.

### 2.3 Assessment of fish oil supplement use

Information about fish oil supplement use was collected through a touch screen questionnaire in which participants were asked, “Do you regularly take any of the following supplements?” Various supplements, including fish oil, were listed in this question for the participants to mark the relevant ones. Data on fish oil supplement use collected at baseline were used for analysis.

### 2.4 Assessment of physical activity

Data on the type (walking, moderate, or vigorous), frequency and duration of PA were obtained from the completed touch screen questionnaires. Furthermore, the metabolic equivalent task (MET) scores were calculated based on the International Physical Activity Questionnaire guidelines. The MET score has been described in detail elsewhere [Guidelines for data processing and analysis of the international physical activity questionnaire (IPAQ)—short and long forms, 2005]. In this study, we assessed the PA types (moderate, vigorous, and walking) using MET minutes per week (MET-minutes/week). The sum of the MET-minutes/week for all three PAs was also calculated (total PA).

### 2.5 Other measurements

Other assessed variables included age (continuous), sex (male or female), ethnicity (White or non-White), TDI, BMI, smoking status (on most or all days, only occasionally, or never), drinking status (current, previous, or never), disease status, and dietary composition. The TDI was used to identify the socio-economic status. It measures regional deprivation derived from national census data on unemployment, car ownership, household overcrowding, and owner’s occupation. Higher scores indicate higher levels of deprivation (Townsend, 1987). BMI was calculated by dividing the weight (kg) by the height squared (m^2^). History of diabetes was defined based on self-reported diabetes at baseline, having been diagnosed with diabetes by a physician, or taking medications to treat diabetes. Cardiovascular diseases (CVD) were defined based on self-reported hypertension, heart problems, cerebrovascular disease, peripheral vascular disease, or other cardiovascular-related diseases. Dietary data were obtained from the touch screen food frequency questionnaire. As in previous studies (Wang et al., 2022), we coded the frequency of various food intakes into scores: never = 0, less than once a week = 0.5, once a week = 1, 2–4 times a week = 3, 5–6 times a week = 5.5, and once or more daily = 7. We then calculated the frequency scores for vegetables, fruit, fish, unprocessed red meat, and processed meat by grouping and summarizing the scores obtained from the above coding rules for each food item.

### 2.6 Polygenic risk score

The polygenic risk score (PRS) shows the correlation between genotype and risk of Parkinson’s disease through a score format. In the present study, we applied the standard PRS for Parkinson’s disease released from the UK Biobank (Field ID: 26260), the calculation of which has been specifically described in the study by Thompson et al. (2022). The study by Thompson et al. calculated polygenic risk scores for 28 diseases and 25 quantitative traits. Standard PRS were generated from an external GWAS meta-analysis dataset and the RPS algorithm was built from trait-specific meta-analyses using Bayesian approach. The PRS value for each individual was calculated as the genome-wide sum of the per-variant posterior effect size multiplied by allele dosage. For the generated raw PRS, centering and standardization steps were followed to generate a corrected PRS for subsequent analysis.

### 2.7 Statistical analysis

Descriptive statistics are expressed as means [standard deviations (SDs)] for continuous variables and numbers (percentages) for categorical variables. Baseline characteristics of participants with and without PD onset during follow-up were compared by analysis of variance (ANOVA) for normally distributed continuous variables, Mann–Whitney U test for non-normally distributed continuous variables, and chi-squared tests for categorical variables.

First, we used the Cox proportional hazards regression model to assess the relationship between PA and PD incidence. Specifically, we analyzed the association between individual PA types (moderate, vigorous, walking, and total) and PD incidence. When the PA types were analyzed as continuous variables, the results are expressed as the association between change per 1,000 MET-minutes/week and PD incidence. We further divided the PAs into four subgroups based on the quartiles to analyze them as categorical variables, using the lowest quartile subgroup as a reference. Second, we used the Cox proportional hazards regression model to analyze the association between fish oil supplement use and PD incidence. Finally, in search of an interaction between fish oil supplement use and PA in affecting PD incidence, we stratified the participants according to their fish oil supplement use and explored the effect of the various PA levels on PD progression. We used likelihood ratio tests to assess for interaction when PA was considered a categorical variable. When PA was considered a continuous variable, we included a cross-product term for PA and fish oil supplement use in the model to test the interaction. Besides, we used restricted cubic spline to further observe the dose-dependent relationship between PA and PD incidence in participants with different fish oil supplement use status. In this study, model 1 was adjusted for age and sex; model 2 was further adjusted for race, TDI, BMI, smoking status, drinking status, CVD, diabetes, and fish, vegetable, fruit, unprocessed red meat, and processed meat intake frequency scores. Model 2 was further adjusted for fish oil supplement use when analyzing the association between PA and PD progression. Model 2 was further adjusted for total PA when analyzing the association between fish oil supplement use and PD progression. The results are expressed as hazard ratios (HRs) and their 95% confidence intervals (CIs).

In addition, we conducted a series of additional analyses. First, considering the possible sex variability of the above associations, we further explored the associations of fish oil supplement use and PA with PD risk in different sex populations. Second, to test whether the effect of fish oil or physical activity on the occurrence of PD differed across population with different PD genetic risk, we examining the interaction effect of genetic risk and fish oil supplement use or physical activity on the risk of PD separately. Populations with high PD genetic risk, Intermediate PD genetic risk and low PD genetic risk were classified by the PD-PRS tertiles. Finally, we performed a sensitivity analysis by excluding participants who developed Parkinson’s disease in the 2 years prior to study follow-up in order to exclude participants whose occurrence of PD might not be related to PA and fish oil supplements use.

Statistical analysis was performed with R software, Version 4.2.1. Two-sided *p* < 0.05 was considered statistically significant.

## 3 Results

### 3.1 Baseline characteristics

The cohort baseline characteristics were presented in Supplementary Table S2. Participants incident PD during the follow up were more likely to be older, male, white, with higher BMI, and a history of CVD and diabetes (*p* < 0.001), compared with participants no incident PD.

### 3.2 Association between fish oil supplement use or physical activity and Parkinson’s disease development

During a median follow-up of 12.52 years, 2,131 participants developed PD. The association between PA or fish oil supplement use and PD incidence was presented in Tables 1, 2. We found that after adjusting for a range of variables, participants using fish oil supplements had a lower risk of developing PD than non-users (HR, 0.89; 95% CI, 0.82–0.98). For each 1,000 MET-minute/week increase, the adjusted HRs for PD occurrence were 0.96 (95% CI, 0.95–0.98) for total PA, 0.93 (95% CI, 0.90–0.96) for moderate PA, 0.95 (95% CI, 0.91–0.99) for vigorous PA, and 0.93 (95% CI, 0.89–0.98) for walking. When the amounts of PA were analyzed as categorical variables, participants with highest PA level (Q4) showed a significantly lower risk of PD occurrence than those with lowest PA level (Q1). The trend analysis showed that the associations between the activity level for all the three types of PA, as well as total PA level and the risk of developing PD showed significant negative linear trends (*P* for trend < 0.001).

**Table 1:** Associations of physical activity with incident PD (*n* = 385,275).

	Number	Model 1		Model 2	
		HR (95%CI)	*p*-value	HR (95%CI)	*p*-value
Physical activity (sum)					
Continues^a^		0.96(0.94,0.98)	**<0.001**	0.96(0.95,0.98)	**<0.001**
Q1 (0,810)	96,534	Ref		Ref	
Q2 (810,1773)	96,449	0.89(0.80,1.00)	0.056	0.91(0.81,1.02)	0.116
Q3 (1773,3,546)	96,094	0.77(0.68,0.87)	**<0.001**	0.79(0.70,0.90)	**<0.001**
Q4 (3,546,19,278)	96,198	0.73(0.65,0.82)	**<0.001**	0.75(0.67,0.85)	**<0.001**
P-trend			**<0.001**		**<0.001**
Physical activity (moderate)					
Continues^a^		0.92(0.89,0.95)	**<0.001**	0.93(0.90,0.96)	**<0.001**
Q1 (0,120)	102,703	Ref		Ref	
Q2 (120,480)	106,907	0.85(0.76,0.95)	**0.005**	0.87(0.78,0.98)	**0.018**
Q3 (480,1,200)	84,736	0.74(0.66,0.84)	**<0.001**	0.76(0.67,0.87)	**<0.001**
Q4 (1,200,5,040)	90,929	0.73(0.65,0.82)	**<0.001**	0.75(0.67,0.85)	**<0.001**
P-trend			**<0.001**		**<0.001**
Physical activity (vigorous)					
Continues^a^		0.94(0.91,0.98)	**0.003**	0.95(0.91,0.99)	**0.01**
Q1 (0,0)	155,914	Ref		Ref	
Q2 (0,240)	53,537	0.94(0.83,1.07)	0.383	0.98(0.86,1.12)	0.793
Q3 (240,960)	96,116	0.93(0.83,1.03)	0.167	0.96(0.86,1.08)	0.513
Q4 (960,10,080)	79,708	0.77(0.68,0.87)	**<0.001**	0.80(0.71,0.91)	**<0.001**
P-trend			**<0.001**		**<0.001**
Physical activity (walking)					
Continues^a^		0.93(0.89,0.97)	**<0.001**	0.93(0.89,0.98)	**0.002**
Q1 (0,297)	97,582	Ref		Ref	
Q2 (297,693)	125,460	0.96(0.86,1.07)	0.459	0.97(0.87,1.08)	0.594
Q3 (693,1,386)	88,853	0.91(0.80,1.02)	0.111	0.92(0.81,1.04)	0.184
Q4 (1,386,4,158)	73,380	0.79(0.69,0.91)	**<0.001**	0.81(0.71,0.93)	**0.002**
P-trend			**<0.001**		**<0.001**

Model 1: Adjusted for age, sex. Model 2: Adjusted for age, sex, race, Townsend deprivation index, BMI, smoke, alcohol, cardiovascular disease, diabetes, fish, fruit, vegetable, processed meat, unprocessed meat, fish oil supplement.

a Continuous expressed as change per 1,000 units.

Bold represents *p*-value <0.05.

**Table 2:** Associations of fish oil supplementation use with incident PD (*n* = 385,275).

	Number	Model 1		Model 2	
		HR (95%CI)	*p*-value	HR (95%CI)	*p*-value
Fish oil supplement use					
No	263,747	Ref		Ref	
Yes	121,528	0.88(0.80,0.96)	**0.004**	0.89(0.82,0.98)	**0.016**

Model 1: Adjusted for age, sex. Model 2: Adjusted for age, sex, race, Townsend deprivation index, BMI, smoke, alcohol, cardiovascular disease, diabetes, fish, fruit, vegetable, processed meat, unprocessed meat, sum of PA. Bold represents *p*-value <0.05.

### 3.3 Joint effect of fish oil supplement use and physical activity on Parkinson’s disease incidence

The association between PA and the occurrence of PD after stratification by fish oil supplement use was shown in Table 3. When analyzing the PA levels as categorical variables, a significant interaction was noted between total or moderate PA levels and fish oil supplement use (*P* for interaction <0.05). The HR of the highest total PA level (Q4), when compared to the lowest PA level (Q1), was 0.62 (95% CI, 0.50–0.76) in the fish oil supplement-using population and 0.84 (95% CI, 0.72–0.97) in the non-users. The HR of the highest moderate PA level (Q4), when compared to the lowest PA level (Q1), was 0.59 (95% CI, 0.48–0.73) for the fish oil supplement-using population and 0.86 (95% CI, 0.74–0.99) for non-users. When PA levels was analyzed as a continuous variable, we found a significant interaction between fish oil supplement use and total PA (*P* for interaction = 0.011), moderate PA (*P* for interaction = 0.015), and walking activity (*P* for interaction = 0.029) in their effect on PD incidence. This finding was reflected in the more significant reduction in PD risk in fish oil supplement users than in non-users with increasing of total, moderate, and walking PA levels.

**Table 3:** Joint associations of fish oil supplementation and PA with incident PD (*n* = 385,275).

	Category					Continues	
	Q1	Q2	Q3	Q4	*P* for interaction	Per1000 Met increase	*P* for interaction
Physical activity (sum)							
Using fish oil supplement							
Yes	Ref	0.83(0.68,1.01) 0.059	**0.63(0.51,0.78) < 0.001**	**0.62(0.50,0.76) < 0.001**	**0.0275**	**0.93(0.90,0.96) < 0.001**	**0.0105**
No	Ref	0.95(0.82,1.10) 0.523	0.89(0.77,1.04) 0.132	**0.84(0.72,0.97) 0.021**		**0.98(0.96,1.00) 0.044**	
Physical activity (walking)							
Using fish oil supplement							
Yes	Ref	0.87(0.72,1.05) 0.134	0.85(0.69,1.04) 0.116	**0.64(0.50,0.81) < 0.001**	0.0683	**0.88(0.81,0.95) < 0.001**	**0.0289**
No	Ref	1.03(0.90,1.18) 0.683	0.96(0.82,1.12) 0.579	0.91(0.77,1.08) 0.293		0.97(0.92,1.02) 0.183	
Physical activity (moderate)							
Using fish oil supplement							
Yes	Ref	0.83(0.68,1.01) 0.058	**0.63(0.51,0.78) < 0.001**	**0.59(0.48,0.73) < 0.001**	**0.0089**	**0.87(0.82,0.93) < 0.001**	**0.0151**
No	Ref	0.88(0.77,1.02) 0.096	**0.84(0.72,0.99) 0.032**	**0.86(0.74,0.99) 0.039**		0.96(0.92,1.00) 0.066	
Physical activity (vigorous)							
Using fish oil supplement							
Yes	Ref	1.01(0.86,1.19) 0.916	1.04(0.91,1.19) 0.539	**0.85(0.73,0.99) 0.041**	0.3278	**0.92(0.86,0.99) 0.017**	0.2631
No	Ref	0.93(0.75,1.16) 0.541	0.84(0.70,1.01) 0.060	**0.72(0.59,0.89) 0.002**		0.97(0.92,1.01) 0.165	

Model: Adjusted for age, sex, race, Townsend deprivation index, BMI, smoke, alcohol, cardiovascular disease, diabetes, fish, fruit, vegetable, processed meat, unprocessed meat. Bold represents *p*-value <0.05.

**Figure 1:**
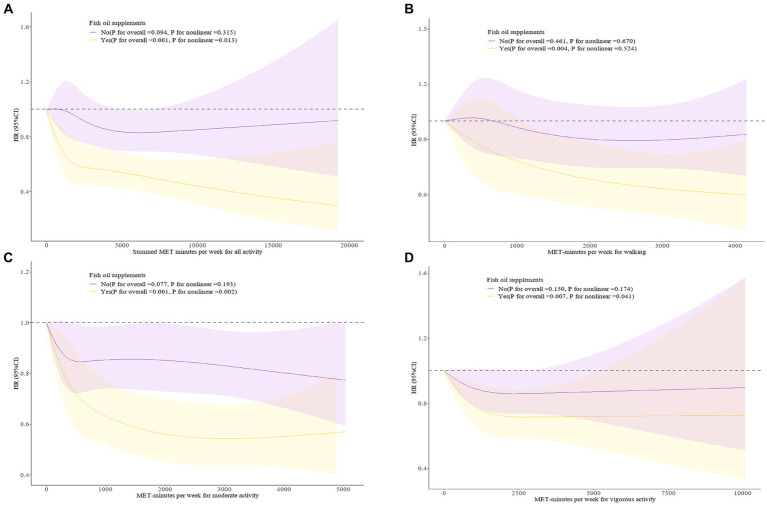
**(A)** Dose–response associations between Summed MET-minutes per week for all activity and fish oil supplement with PD incidence. **(B)** Dose–response associations between MET minutes per week for walking and fish oil supplement with PD incidence. **(C)** Dose–response associations between MET minutes per week for moderate activity and fish oil supplement with PD incidence. **(D)** Dose–response associations between MET minutes per week for vigorous activity and fish oil supplement with PD incidence. The analysis was performed after adjusting for age, sex, race, Townsend deprivation index, BMI, smoke, alcohol, cardiovascular disease, diabetes, fish, fruit, vegetable, processed meat, unprocessed meat. 95%CI, 95% confidence interval; HR, hazard ratio; MET, metabolic equivalent task.

Restricted cubic splines (Figure 1) examined the dose–response associations between the PA levels and PD occurrence in populations with different fish oil supplement use statuses. We found no significant association between levels of various types of PA and PD risk in the group not using fish oil supplements (*P* for overall > 0.05). In those using fish oil supplements, for total PA (*P* for overall < 0.001), and walking (*P* for overall = 0.004), the risk of PD continued to decrease with increasing PA levels. For vigorous PA, we found a significant negative association between lower vigorous PA levels and PD occurrence in fish oil supplement users. However, this association reversed to a positive correlation and lost statistical significance above a certain PA level (*P* for overall = 0.007, *P* for nonlinear = 0.041). In the population using fish oil supplements, moderate PA levels were found to be “U-shaped” associated with the risk of PD (*P* for overall < 0.001, *P* for nonlinear = 0.002).

### 3.4 Sex differences in the joint effect of fish oil supplement use and physical activity on Parkinson’s disease

Baseline characteristics for males and females were detailed in Supplementary Tables S3, S4. Supplementary Table S5 shows the association between PA and PD occurrence after stratification by fish oil supplement use in males. We found a significant interaction between the total, walking, and moderate PA levels and fish oil supplement use when PA level was analysed as categorical variables (*P* for interaction < 0.05). The HR for the highest total PA level (Q4), when compared to the lowest level (Q1), was 0.55 (95% CI, 0.42–0.71) in fish oil supplement users and 0.84 (95% CI, 0.69–1.01) in non-users. The HR for the highest moderate PA level (Q4), when compared to the lowest level (Q1), was 0.51 (95% CI, 0.39–0.66) for fish oil supplement users and 0.82 (95% CI, 0.68–0.99) for non-users. The HR for the highest walking PA level (Q4), when compared to the lowest level (Q1), was 0.56 (95% CI, 0.42–0.75) for fish oil supplement users and 0.92 (95% CI, 0.75–1.13) for non-users. When the PA level was analyzed as a continuous variable, we also found a significant interaction between fish oil supplement use and total PA (*P* for interaction = 0.0039), moderate PA (for interaction *p* = 0.0028), and walking activity (for interaction *p* = 0.0342) in their effect on the incidence of PD.

Supplementary Table S6 shows the association between PA and PD occurrence after stratification by fish oil supplement use in females. No interaction was found between categorical total, moderate, vigorous, and walking PA levels and fish oil supplement use (*P* for interaction >0.05).

Restricted cubic splines examined the dose–response relationship between PA levels and PD incidence in males and females with various fish oil supplement use statuses (Supplementary Figure S2). The dose–response relationship between the PA level and PD incidence in males with various fish oil supplement statuses resembled that of the total population. In contrast, in the female population, no significant associations were observed for PA levels and PD risk in either those using or not using fish oil supplements (*P* for overall >0.05).

### 3.5 Association of fish oil supplementation use or PA with PD risk in participants with different genetic susceptibilities for PD

In this analysis, we did not find a significant interaction between fish oil supplementation use and genetic risk on PD risk (for interaction *p* = 0.2701) (Supplementary Table S7). Also, we did not find a significant interaction between PA and genetic risk on PD risk (for interaction *p* > 0.05) (Supplementary Table S8).

### 3.6 Sensitivity analysis

We further analyzed the cohort after excluding participants who developed PD within 2 years of study. The combined effect of fish oil supplement use and PA on the risk of PD, both in the total population and in the male as well as female populations, was mostly consistent with the results observed in the entire cohort (Supplementary Tables S9–S11).

## 4 Discussion

This prospective cohort study of 385,275 participants in the United Kingdom found that fish oil supplement use and all PA intensities (total, moderate, vigorous, and walking) were associated with a lower risk of developing PD. Notably, we found a significant interaction between PA levels and fish oil supplement use, with fish oil supplement use enhancing the effect of PA on PD risk. This effect was more pronounced in males than females. In addition, based on the interaction test, we observed that PD genetic risk did not affect the effect of fish oil supplementation use or PA on PD incidence.

Consistent with previous studies (Abbott et al., 2003; de Lau et al., 2005; Gao et al., 2007; Denny Joseph and Muralidhara, 2015; Hernando et al., 2019), our study found a similarly strong association between fish oil supplement use and a reduced risk of PD. Fish oil supplements, particularly omega-3 polyunsaturated fatty acids, are essential lipid nutrients in the human diet and play a key role in cell membrane structure. Omega-3 fatty acids have been shown to inhibit microglial cell activity and neuroinflammation, protect astrocyte function, and produce neurotrophic factors that improve neurodegeneration and normalize neurotransmission (McCarty and Lerner, 2020). Recent studies have also shown that omega-3 fatty acids improve PD by inhibiting pro-inflammatory cytokine release, restoring mitochondrial function and membrane fluidity, and reducing levels of oxidant production (Wu et al., 2021). The omega-3 fatty acid docosahexaenoic acid increases dopamine synthesis in striatal motor areas by phosphorylating the restrictive catecholamine synthase tyrosine hydroxylase in a manner dependent on second messenger-linked protein kinases (PKA and PKC), thus preventing deficits in postural stability, gait integrity, and dopamine neurochemistry (Chitre et al., 2020).

PA protection against PD was first suggested in 1992 (Sasco et al., 1992). Those authors found that increasing levels of PA were associated with a progressively lower risk of PD. Since that initial report, several subsequent epidemiological studies have confirmed this putative relationship (Kyrozis et al., 2013). Our findings are consistent with theirs. A meta-analysis of over half a million adults showed that a higher PA level—particularly moderate to vigorous PA—was associated with a lower risk of PD (Fang et al., 2018). While the importance of moderate to vigorous PA in reducing the risk of PD is widely recognized, little is known about its impact on walking activity. To our knowledge, this was the first study to directly support the protective effect of walking activity on PD development in the general population. In addition, the results of the Cox proportional hazards model revealed that the risk of PD was significantly lower in the group with moderate physical activity above the lowest quartile (120 MET-min/week) compared to the group with moderate physical activity below the lowest quartile. Therefore, adults with limited self-directed time can benefit from a protective effect by choosing moderate exercise at a reduced duration. Several mechanisms have been proposed to explain the neuroprotective effects of PA. For example, PA has been shown to upregulate the production of various growth factors and receptors, maintain dopaminergic function, and reduce cellular inflammation and oxidative stress in animal models of PD (LaHue et al., 2016). Moreover, PA reduces damage to dopaminergic neurons in motor circuits, preserves striatal dopamine levels after treadmill activity in a rodent model of lesion-induced PD, and increases the loss of dopaminergic neurons after forced non-use of the contralateral forelimb (Tillerson et al., 2002).

This study was the first to prospectively and systematically examine the joint association of fish oil supplement use and PA with PD incidence. By using a longitudinal design, fish oil supplement use and PA measurements, and a large sample, this study provided direct and strong evidence of this association. Our findings showed a significant interaction between PA and fish oil supplement use, suggesting that fish oil supplement use improves the protection against and prevention of PD incidence by PA. Increasing the PA level resulted in a significantly lower PD incidence in fish oil supplement users than non-users. At certain activity levels, a significant association between PA and PD was observed only in those using fish oil supplements. These results emphasize the importance of promoting public health strategies of any PA intensity to combat the risk of PD. Given the high prevalence of PD, interventions that include adequate fish oil supplement intake should be particularly emphasized and encouraged for physically inactive people. Protection can also be achieved by consuming fish oil supplements and performing low-intensity or low-frequency PA. Those tolerating high-intensity or high-frequency exercise can be protected even more by consuming fish oil supplements. In summary, the current study highlights the potential benefits of combining fish oil supplement use and PA in PD prevention.

Interestingly, we also found that the combined association of fish oil supplement use and PA with PD incidence differed among the sexes. Our findings showed that males increased the protective effect of PA on PD incidence by consuming fish oil supplements, while females did not. Previous studies have shown a significant association between PA and reduced PD incidence in males but not females (Llamas-Velasco et al., 2021). Furthermore, sex also affects the responsiveness to omega-3 fatty acid supplementation, with higher increases in plasma docosahexaenoic acid and lower triglycerides in males than females (Caslake et al., 2008; Danthiir et al., 2018). A meta-analysis showed that lower total cholesterol, triglycerides, high-density lipoprotein cholesterol, and low-density lipoprotein cholesterol levels were associated with PD development (Hong et al., 2022). Maybe it would help to suggest why it happens in males and not females. Our study had multiple strengths. The combined relationship between fish oil supplements and PA and PD onset has been inadequately studied. Our study provided new insights into this joint relationship. The study was based on a prospective cohort from the UK Biobank. This cohort is well-suited for studying exposure-disease relationships because of its large size, long follow-up, detailed and comprehensive information.

Our study had several limitations that should be acknowledged. First, although the questionnaire is widely used to quantify PA, it is a self-report measure that might be subject to reporting bias. Second, the dietary information in the UK Biobank study is self-reported, limited in scope, and might not provide a thorough picture of the overall healthy eating behavior. Third, despite controlling for multiple covariates, residual confounders cannot be completely excluded. Fourth, this study was conducted based on UK Biobank, the main participants of which were from a white ethnic background, so further research is still needed to validate the applicability of the findings of this study to other ethnic populations. Finally, although UK Biobank recruited a sample over 500,000, it actually had a low response rate (5.5%) and there may have been selection bias (Allen et al., 2012). However, by comparison with other studies, risk factor associations in UK Biobank seem to be generalizable (Batty et al., 2020). Future studies, e.g., clinical trials, are needed to confirm and determine the causal relationship of the associations observed in our study.

## 5 Conclusion

In this UK population-based study, we found that fish oil supplementation and PA reduce the incidence of PD, irrespective of genetic risk. Besides, fish oil supplement use additionally improved the protective effect of PA against PD incidence.

## Data availability statement

Publicly available datasets were analyzed in this study. This data can be found here: the study protocol is publicly available on the UK Biobank website (http://www.ukbiobank.ac.uk/).

## Ethics statement

The studies involving humans were approved by the North West Multi-Centre Research Ethics Committee approved the UK Biobank study, and all participants provided written informed consent to participate. The study protocol is publicly available on the UK Biobank website (http://www.ukbiobank.ac.uk/). The studies were conducted in accordance with the local legislation and institutional requirements. The participants provided their written informed consent to participate in this study.

## Author contributions

FL: Conceptualization, Data curation, Formal analysis, Methodology, Writing – original draft, Visualization. YS: Conceptualization, Data curation, Formal analysis, Methodology, Visualization, Writing – original draft. JZ: Formal analysis, Methodology, Writing – original draft. YL: Data curation, Investigation, Writing – original draft. XuaC: Data curation, Software, Writing – original draft. XZ: Writing – review & editing. YH: Conceptualization, Writing – original draft. KC: Writing – review & editing. YZ: Writing – review & editing. QY: Writing – review & editing. XiaC: Writing – review & editing, Supervision. XinC: Writing – review & editing, Supervision. YW: Writing – review & editing, Supervision. GC: Writing – review & editing, Supervision, Funding acquisition, Resources.

## Footnotes

^1^ http://www.ukbiobank.ac.uk/
