# Supplementary material for: Fish oil supplementation, physical activity and risk of incident Parkinson’s disease: results of longitudinal analysis from the UK Biobank
Source: Front Aging Neurosci. 2024 Jan 29;15:1304629. doi: 10.3389/fnagi.2023.1304629 (PMC10859434; doi:10.3389/fnagi.2023.1304629)
Supplement: Supplementary file 1 [file Data_Sheet_1.docx]

**Table S1** Parkinson’s disease code lists.

| UK Biobank Self Report Codes | | |
| --- | --- | --- |
| Code Type | Code | Biobank Code Text |
| UK Biobank Self Report | Field 20002  Code 1262 | Parkinson's disease |
| ICD 9 Code | | |
| Code Type | ICD 9 Code | ICD 9 Text |
| ICD 9 Code | 3320 | Paralysis agitans |
| ICD 10 Code | | |
| Code Type | ICD 10 Code | ICD 10 Text |
| ICD 10 Code | G20 | Parkinson’s disease |

**Table S2.** Baseline characteristics of no incident Parkinson's disease and incident Parkinson's disease(n=385275).

| Variables | Overall | No incident Parkinson's disease | Incident Parkinson's disease | *P*-value |
| --- | --- | --- | --- | --- |
|  | 385275 | 383144 | 2131 |  |
| Age at baseline (mean (SD)) | 56.27 (8.11) | 56.23 (8.10) | 63.06 (5.23) | <0.001 |
| Sex (%) |  |  |  | <0.001 |
| Female | 203018 (52.7) | 202276 (52.8) | 742 (34.8) |  |
| Male | 182257 (47.3) | 180868 (47.2) | 1389 (65.2) |  |
| Body mass index (mean (SD)) | 27.29 (4.70) | 27.29 (4.70) | 27.69 (4.49) | <0.001 |
| Townsend deprivation index (mean (SD)) | -1.43 (3.01) | -1.43 (3.01) | -1.49 (2.98) | 0.388 |
| Diabetes (%) |  |  |  | <0.001 |
| No | 366078 (95.0) | 364169 (95.0) | 1909 (89.6) |  |
| Yes | 19197 (5.0) | 18975 (5.0) | 222 (10.4) |  |
| Cardiovascular disease (%) |  |  |  | <0.001 |
| No | 248489 (64.5) | 247515 (64.6) | 974 (45.7) |  |
| Yes | 136786 (35.5) | 135629 (35.4) | 1157 (54.3) |  |
| Race (%) |  |  |  | <0.001 |
| Others | 17849 (4.6) | 17785 (4.6) | 64 (3.0) |  |
| White | 367426 (95.4) | 365359 (95.4) | 2067 (97.0) |  |
| Fruit (mean (SD)) | 3.11 (2.57) | 3.11 (2.57) | 3.31 (2.61) | <0.001 |
| Vegetable (mean (SD)) | 4.94 (3.35) | 4.94 (3.35) | 4.99 (3.37) | 0.472 |
| Fish (mean (SD)) | 2.29 (1.59) | 2.28 (1.59) | 2.41 (1.64) | <0.001 |
| Unprocessed meat (mean (SD)) | 2.11 (1.43) | 2.11 (1.43) | 2.16 (1.45) | 0.111 |
| Processed meat (mean (SD)) | 1.48 (1.39) | 1.48 (1.39) | 1.54 (1.41) | 0.05 |
| Current smoking status (%) |  |  |  |  |
| No | 346630 (90.0) | 344627 (89.9) | 2003 (94.0) | <0.001 |
| Only occasionally | 10604 (2.8) | 10574 (2.8) | 30 (1.4) |  |
| Yes, on most or all days | 28041 (7.3) | 27943 (7.3) | 98 (4.6) |  |
| Alcohol status (%) |  |  |  |  |
| Current | 357675 (92.8) | 355769 (92.9) | 1906 (89.4) | <0.001 |
| Never | 14675 (3.8) | 14564 (3.8) | 111 (5.2) |  |
| Previous | 12925 (3.4) | 12811 (3.3) | 114 (5.3) |  |
| Fish oil supplementation (%) |  |  |  |  |
| No | 263747 (68.5) | 262366 (68.5) | 1381 (64.8) | <0.001 |
| Yes | 121528 (31.5) | 120778 (31.5) | 750 (35.2) |  |
| Sum MET minutes per week for all activity | 1773.00 [810.00, 3546.00] | 1773.00 [810.00, 3546.00] | 1626.00 [742.25, 3367.75] | 0.002 |
| MET minutes per week for moderate activity | 480.00 [120.00, 1200.00] | 480.00 [120.00, 1200.00] | 480.00 [120.00, 1200.00] | 0.302 |
| MET minutes per week for vigorous activity | 240.00 [0.00, 960.00] | 240.00 [0.00, 960.00] | 160.00 [0.00, 720.00] | <0.001 |
| MET minutes per week for walking | 693.00 [297.00, 1386.00] | 693.00 [297.00, 1386.00] | 660.00 [297.00, 1386.00] | 0.074 |

The normally distributed continuous variable was represented as mean (standard deviation), the non-normally distributed continuous variable was represented as median [IQR], and categorical variables was represented as n (%).

Abbreviation: MET, metabolic equivalent task; IQR, interquartile range.

**Table S3** Baseline characteristics of no incident Parkinson's disease and incident in male (n = 182257)

| Variables | Overall | No incident Parkinson's disease | Incident Parkinson's disease | *P*-value |
| --- | --- | --- | --- | --- |
|  | 182257 | 180868 | 1389 |  |
| Age at baseline (mean (SD)) | 56.63 (8.19) | 56.58 (8.18) | 63.27 (5.12) | <0.001 |
| Body mass index (mean (SD)) | 27.75 (4.18) | 27.75 (4.18) | 27.80 (4.15) | 0.717 |
| Townsend deprivation index (mean (SD)) | -1.43 (3.06) | -1.43 (3.06) | -1.51 (3.03) | 0.37 |
| Diabetes (%) |  |  |  | <0.001 |
| No | 170164 (93.4) | 168952 (93.4) | 1212 (87.3) |  |
| Yes | 12093 (6.6) | 11916 (6.6) | 177 (12.7) |  |
| Cardiovascular disease (%) |  |  |  | <0.001 |
| No | 108203 (59.4) | 107592 (59.5) | 611 (44.0) |  |
| Yes | 74054 (40.6) | 73276 (40.5) | 778 (56.0) |  |
| Race (%) |  |  |  | 0.001 |
| Others | 8121 (4.5) | 8084 (4.5) | 37 (2.7) |  |
| White | 174136 (95.5) | 172784 (95.5) | 1352 (97.3) |  |
| Fruit (mean (SD)) | 2.80 (2.57) | 2.80 (2.57) | 3.06 (2.48) | <0.001 |
| Vegetable (mean (SD)) | 4.71 (3.45) | 4.71 (3.45) | 4.90 (3.47) | 0.044 |
| Fish (mean (SD)) | 2.26 (1.60) | 2.26 (1.60) | 2.39 (1.65) | 0.002 |
| Unprocessed meat (mean (SD)) | 2.27 (1.49) | 2.27 (1.49) | 2.26 (1.48) | 0.662 |
| Processed meat (mean (SD)) | 1.87 (1.51) | 1.88 (1.51) | 1.78 (1.48) | 0.023 |
| Smoking status (%) |  |  |  | <0.001 |
| No | 160888 (88.3) | 159584 (88.2) | 1304 (93.9) |  |
| Only occasionally | 6304(3.5) | 6284(3.5) | 20(1.4) |  |
| Yes, on most or all days | 15065(8.3) | 15000(8.3) | 65(4.7) |  |
| Alcohol status (%) |  |  |  | <0.001 |
| Current | 172003 (94.4) | 170728 (94.4) | 1275 (91.8) |  |
| Never | 4293(2.4) | 4250(2.3) | 43(3.1) |  |
| Previous | 5961(3.3) | 5890(3.3) | 71(5.1) |  |
| Fish oil supplementation (%) |  |  |  | 0.001 |
| No | 127313 (69.9) | 126399 (69.9) | 914 (65.8) |  |
| Yes | 54944 (30.1) | 54469 (30.1) | 475 (34.2) |  |
| Sum MET minutes per week for all activity | 1830.00 [813.00, 3732.00] | 1832.00 [813.00, 3732.00] | 1596.00 [735.00, 3367.50] | <0.001 |
| MET minutes per week for moderate activity | 480.00 [120.00, 1200.00] | 480.00 [120.00, 1200.00] | 420.00 [120.00, 1200.00] | 0.071 |
| MET minutes per week for vigorous activity | 240.00 [0.00, 960.00] | 240.00 [0.00, 960.00] | 160.00 [0.00, 720.00] | <0.001 |
| MET minutes per week for walking | 693.00 [297.00, 1386.00] | 693.00 [297.00, 1386.00] | 660.00 [297.00, 1386.00] | 0.278 |

The normally distributed continuous variable was represented as mean (standard deviation), the non-normally distributed continuous variable was represented as median [IQR], and categorical variables was represented as n (%).

Abbreviation: MET, metabolic equivalent task; IQR, interquartile range.

**Table S4** Baseline characteristics of no incident Parkinson's disease and incident Parkinson's disease in female (n=203018).

| Variables | Overall | No incident Parkinson's disease | Incident Parkinson's disease | *P*-value |
| --- | --- | --- | --- | --- |
|  | 203018 | 202276 | 742 |  |
| Age at baseline (mean (SD)) | 55.95 (8.02) | 55.93 (8.02) | 62.68 (5.41) | <0.001 |
| Body mass index (mean (SD)) | 26.87 (5.08) | 26.87 (5.08) | 27.50 (5.08) | 0.001 |
| Townsend deprivation index (mean (SD)) | -1.44 (2.97) | -1.44 (2.97) | -1.46 (2.88) | 0.815 |
| Diabetes (%) |  |  |  | <0.001 |
| No | 195914 (96.5) | 195217 (96.5) | 697 (93.9) |  |
| Yes | 7104 (3.5) | 7059 (3.5) | 45 (6.1) |  |
| Cardiovascular disease (%) |  |  |  | <0.001 |
| No | 140286 (69.1) | 139923 (69.2) | 363 (48.9) |  |
| Yes | 62732 (30.9) | 62353 (30.8) | 379 (51.1) |  |
| Race (%) |  |  |  | 0.165 |
| Others | 9728 (4.8) | 9701 (4.8) | 27 (3.6) |  |
| White | 193290 (95.2) | 192575 (95.2) | 715 (96.4) |  |
| Fruit (mean (SD)) | 3.39 (2.54) | 3.39 (2.54) | 3.76 (2.76) | <0.001 |
| Vegetable (mean (SD)) | 5.15 (3.24) | 5.15 (3.24) | 5.18 (3.15) | 0.822 |
| Fish (mean (SD)) | 2.31 (1.59) | 2.31 (1.59) | 2.45 (1.63) | 0.015 |
| Unprocessed meat (mean (SD)) | 1.96 (1.35) | 1.96 (1.35) | 1.98 (1.37) | 0.783 |
| Processed meat (mean (SD)) | 1.12 (1.16) | 1.12 (1.16) | 1.08 (1.13) | 0.294 |
| Smoking status (%) |  |  |  | 0.029 |
| No | 185742 (91.5) | 185043 (91.5) | 699 (94.2) |  |
| Only occasionally | 4300 (2.1) | 4290 (2.1) | 10 (1.3) |  |
| Yes, on most or all days | 12976 (6.4) | 12943 (6.4) | 33 (4.4) |  |
| Alcohol status (%) |  |  |  | <0.001 |
| Current | 185672 (91.5) | 185041 (91.5) | 631 (85.0) |  |
| Never | 10382 (5.1) | 10314 (5.1) | 68 (9.2) |  |
| Previous | 6964 (3.4) | 6921 (3.4) | 43 (5.8) |  |
| Fish oil supplementation (%) |  |  |  | 0.015 |
| No | 136434 (67.2) | 135967 (67.2) | 467 (62.9) |  |
| Yes | 66584 (32.8) | 66309 (32.8) | 275 (37.1) |  |
| Sum MET minutes per week for all activity | 1732.50 [802.00, 3390.00] | 1732.50 [802.50, 3390.00] | 1659.50 [762.75, 3370.50] | 0.289 |
| MET minutes per week for moderate activity | 480.00 [120.00, 1200.00] | 480.00 [120.00, 1200.00] | 480.00 [120.00, 1440.00] | 0.402 |
| MET minutes per week for vigorous activity | 160.00 [0.00, 720.00] | 160.00 [0.00, 720.00] | 0.00 [0.00, 480.00] | <0.001 |
| MET minutes per week for walking | 693.00 [330.00, 1386.00] | 693.00 [330.00, 1386.00] | 594.00 [330.00, 1386.00] | 0.219 |
|  |  |  |  |  |

The normally distributed continuous variable was represented as mean (standard deviation), the non-normally distributed continuous variable was represented as median [IQR], and categorical variables was represented as n (%).

Abbreviation: MET, metabolic equivalent task; IQR, interquartile range.

**Table S5**. Joint associations of fish oil supplementation use and PA with incident PD in male (n=182257)

|  | Category | | | | | Continues | |
| --- | --- | --- | --- | --- | --- | --- | --- |
|  | Q1 | Q2 | Q3 | Q4 | P for interaction | Per1000 Met increase | P for interaction |
|  | Physical activity (Sum) | | | | |  |  |
| Using fish oil supplement |  |  |  |  |  |  |  |
| Yes | Ref | 0.85(0.67,1.09) 0.206 | **0.61(0.47,0.79) <0.001** | **0.55(0.42,0.71) <0.001** | **0.0222** | **0.91(0.88,0.95) <0.001** | **0.0039** |
| No | Ref | 0.98(0.82,1.17) 0.821 | 0.87(0.72,1.04) 0.129 | 0.84(0.69,1.01) 0.060 |  | **0.98(0.95,1.00) 0.065** |  |
| Using fish oil supplement | Physical activity (Walking) | | | | |  |  |
| Yes | Ref | **0.74(0.58,0.94) 0.012** | 0.83(0.64,1.06) 0.129 | **0.56(0.42,0.75) <0.001** | **0.0158** | **0.85(0.78,0.94) 0.001** | **0.0342** |
| No | Ref | 1.04(0.88,1.24) 0.630 | 1.02(0.85,1.23) 0.841 | 0.92(0.75,1.13) 0.418 |  | 0.96(0.90,1.02) 0.196 |  |
| Using fish oil supplement | Physical activity (Moderate) | | | | |  |  |
| Yes | Ref | 0.88(0.69,1.11) 0.280 | **0.62(0.47,0.80) <0.001** | **0.51(0.39,0.66) <0.001** | **0.0018** | **0.82(0.75,0.89) <0.001** | **0.0028** |
| No | Ref | 0.90(0.75,1.07) 0.226 | 0.91(0.76,1.10) 0.324 | **0.82(0.68,0.99) 0.036** |  | 0.95(0.90,1.00) 0.066 |  |
| Using fish oil supplement | Physical activity (Vigorous) | | | | |  |  |
| Yes | Ref | 0.97(0.74,1.27) 0.814 | 0.85(0.67,1.07) 0.163 | **0.68(0.52,0.87) 0.003** | 0.243 | **0.91(0.84,0.98) 0.019** | 0.1587 |
| No | Ref | 1.09(0.90,1.33) 0.368 | 1.08(0.92,1.28) 0.347 | 0.89(0.74,1.07) 0.215 |  | 0.97(0.92,1.02) 0.273 |  |

Model：Adjusted for age, sex, race, Townsend deprivation index, BMI, smoke, alcohol, CVD, DM, fish, fruit, vegetable, processed meat, unprocessed meat.

**Table S6**. Joint associations of fish oil supplementation use and PA with incident PD in female (n=203018)

|  | Category | | | | | Continues | |
| --- | --- | --- | --- | --- | --- | --- | --- |
|  | Q1 | Q2 | Q3 | Q4 | P for interaction | Per1000 Met increase | P for interaction |
|  | Physical activity (Sum) | | | | |  |  |
| Using fish oil supplement |  |  |  |  |  |  |  |
| Yes | Ref | 0.78(0.56,1.09) 0.139 | **0.67(0.47,0.94) 0.021** | 0.75(0.54,1.04) 0.089 | 0.5166 | 0.97(0.92,1.02) 0.179 | 0.6878 |
| No | Ref | 0.90(0.70,1.16) 0.416 | 0.94(0.73,1.20) 0.606 | 0.84(0.64,1.09) 0.182 |  | 0.98(0.95,1.02) 0.380 |  |
| Using fish oil supplement | Physical activity (Walking) | | | | |  |  |
| Yes | Ref | 1.14(0.83,1.57) 0.427 | 0.91(0.63,1.30) 0.593 | 0.82(0.55,1.22) 0.335 | 0.7718 | 0.92(0.81,1.04) 0.159 | 0.4016 |
| No | Ref | 1.00(0.79,1.27) 0.999 | 0.84(0.64,1.10) 0.202 | 0.90(0.68,1.20) 0.485 |  | 0.98(0.89,1.07) 0.615 |  |
| Using fish oil supplement | Physical activity (Moderate) | | | | |  |  |
| Yes | Ref | 0.74(0.53,1.04) 0.079 | **0.65(0.46,0.93) 0.017** | 0.74(0.53,1.02) 0.070 | 0.8558 | 0.96(0.87,1.05) 0.373 | 0.8389 |
| No | Ref | 0.86(0.67,1.10) 0.227 | **0.72(0.55,0.95) 0.020** | 0.92(0.72,1.18) 0.513 |  | 0.98(0.91,1.06) 0.582 |  |
| Using fish oil supplement | Physical activity (Vigorous) | | | | |  |  |
| Yes | Ref | 0.88(0.61,1.26) 0.476 | 0.82(0.60,1.11) 0.190 | 0.83(0.59,1.17) 0.292 | 0.7357 | 0.95(0.84,1.08) 0.466 | 0.8796 |
| No | Ref | 0.85(0.64,1.14) 0.280 | 0.97(0.77,1.23) 0.829 | 0.77(0.57,1.03) 0.075 |  | 0.95(0.86,1.05) 0.343 |  |

Model：Adjusted for age, sex, race, Townsend deprivation index, BMI, smoke, alcohol, cardiovascular disease, diabetes, fish, fruit, vegetable, processed meat, unprocessed meat.

**Table S7.** Association of fish oil supplement use with PD in populations with different genetic risk of PD (n = 376415).

|  | PD-PRS (HR [95% CI], *P*-value) | | | *P* for interaction |
| --- | --- | --- | --- | --- |
|  | Low | Intermediate | High |  |
| No | Ref. | Ref. | Ref. | 0.2701 |
| Yes | 0.85(0.70,1.03) 0.099 | 0.82(0.70,0.97) 0.020 | 0.96(0.84,1.09) 0.516 |  |

*P* value for the interaction between fish oil supplement use and polygenic risk score categories.

Model adjusted for age, sex, race, Townsend deprivation index, BMI, smoke, drink, cardiovascular disease, diabetes, fish, fruit, vegetable, processed meat, unprocessed meat, sum of physical activity.

**Table S8**. Association of physical activity (per 1000 MET-minutes/week) with PD with different genetic risk of PD (n = 376415).

|  | PD-PRS | | |  |
| --- | --- | --- | --- | --- |
|  | Low | Intermediate | High | P for interaction |
| Vigorous | 0.94(0.86,1.02) 0.132 | 0.96(0.90,1.03) 0.259 | 0.93(0.88,0.99) 0.023 | 0.844 |
| Moderate | 0.91(0.84,0.99) 0.020 | 0.95(0.89,1.02) 0.143 | 0.97(0.85,1.11) 0.647 | 0.577 |
| Walking | 0.87(0.78,0.96) 0.004 | 0.98(0.91,1.06) 0.647 | 0.94(0.88,1.00) 0.048 | 0.097 |

*P* value for the interaction between PA and polygenic risk score categories.

Model adjusted for age, sex, race, Townsend deprivation index, BMI, smoke, drink, cardiovascular disease, diabetes, fish, fruit, vegetable, processed meat, unprocessed meat, and fish oil supplement use.

**Table S9**. Joint associations of fish oil supplementation and PA with incident PD after excluding participants who developed PD within the first two years of follow-up (n=385215)

|  | Category | | | | | | Continues | |
| --- | --- | --- | --- | --- | --- | --- | --- | --- |
|  | Q1 | Q2 | | Q3 | Q4 | P for interaction | Per1000 Met increase | P for interaction |
|  | Physical activity (Sum) | | | | | |  |  |
| Using fish oil supplement |  | |  |  |  |  |  |  |
| Yes | Ref | | **0.82(0.67,1.00) 0.050** | **0.63(0.51,0.78) <0.001** | **0.62(0.50,0.76) <0.001** | **0.0209** | **0.93(0.90,0.96) <0.001** | **0.0083** |
| No | Ref | | 0.97 (0.84,1.12) 0.689 | 0.91(0.78,1.06) 0.228 | **0.85(0.73,0.99) 0.041** |  | 0.98(0.96,1.00) 0.065 |  |
| Using fish oil supplement | Physical activity (Walking) | | | | | |  |  |
| Yes | Ref | | 0.85(0.70,1.03) 0.104 | 0.85(0.69,1.05) 0.126 | **0.64(0.50,0.81) <0.001** | 0.0599 | **0.88(0.82,0.95) 0.001** | **0.0294** |
| No | Ref | | 1.04(0.90,1.20) 0.581 | 0.99(0.84,1.15) 0.852 | 0.92(0.78,1.09) 0.350 |  | 0.97(0.92,1.02) 0.234 |  |
| Using fish oil supplement | Physical activity (Moderate) | | | | | |  |  |
| Yes | Ref | | **0.82(0.67,1.00) 0.046** | **0.63(0.51,0.78) <0.001** | **0.59(0.48,0.73) <0.001** | **0.0119** | **0.88(0.82,0.94) <0.001** | **0.0198** |
| No | Ref | | 0.89(0.77,1.03) 0.122 | **0.85(0.72,0.99) 0.036** | **0.86(0.74,1.00) 0.052** |  | 0.96(0.92,1.00) 0.081 |  |
| Using fish oil supplement | Physical activity (Vigorous) | | | | | |  |  |
| Yes | Ref | | 0.95(0.76,1.18) 0.652 | **0.82(0.68,0.99) 0.042** | **0.72(0.58,0.88) 0.002** | 0.1944 | **0.91(0.85,0.98) 0.010** | 0.1609 |
| No | Ref | | 1.02(0.86,1.20) 0.835 | 1.07(0.93,1.22) 0.350 | **0.86(0.73,1.01) 0.063** |  | 0.97(0.92,1.02) 0.217 |  |

Model：Adjusted for age, sex, race, Townsend deprivation index, BMI, smoke, alcohol, cardiovascular disease, diabetes, fish, fruit, vegetable, processed meat, unprocessed meat.

**Table S10**. Joint associations of fish oil supplementation and PA with incident PD in male after excluding participants who developed PD within two years of study(n=182219)

|  | Category | | | | | Continues | |
| --- | --- | --- | --- | --- | --- | --- | --- |
|  | Q1 | Q2 | Q3 | Q4 | P for interaction | Per1000 Met increase | P for interaction |
|  | Physical activity (Sum) | | | | |  |  |
| Using fish oil supplement |  |  |  |  |  |  |  |
| Yes | Ref | 0.85(0.66,1.08) 0.186 | **0.62(0.48,0.80) <0.001** | **0.53(0.41,0.70) <0.001** | **0.0169** | **0.91(0.88,0.95) <0.001** | **0.0016** |
| No | Ref | 0.98(0.82,1.18) 0.851 | 0.87(0.72,1.06) 0.161 | 0.85(0.70,1.02) 0.080 |  | **0.98(0.95,1.00) 0.096** |  |
| Using fish oil supplement | Physical activity (Walking) | | | | |  |  |
| Yes | Ref | **0.75(0.59,0.95) 0.017** | 0.82(0.64,1.06) 0.133 | **0.56(0.42,0.75) <0.001** | **0.0215** | **0.86(0.78,0.94) 0.002** | **0.0280** |
| No | Ref | 1.04(0.87,1.24) 0.674 | 1.04(0.86,1.26) 0.669 | 0.92(0.75,1.13) 0.422 |  | 0.96(0.90,1.03) 0.257 |  |
| Using fish oil supplement | Physical activity (Moderate) | | | | |  |  |
| Yes | Ref | 0.88(0.69,1.12) 0.291 | **0.63(0.48,0.82) 0.001** | **0.50(0.38,0.66) <0.001** | **0.0022** | **0.82(0.75,0.90) <0.001** | **0.0021** |
| No | Ref | 0.90(0.75,1.07) 0.238 | 0.90(0.75,1.09) 0.288 | **0.83(0.69,0.99) 0.043** |  | 0.95(0.90,1.01) 0.088 |  |
| Using fish oil supplement | Physical activity (Vigorous) | | | | |  |  |
| Yes | Ref | 0.99(0.75,1.30) 0.928 | 0.84(0.67,1.07) 0.157 | **0.65(0.50,0.85) 0.001** | 0.1618 | **0.89(0.82,0.97) 0.008** | 0.0690 |
| No | Ref | 1.11(0.91,1.35) 0.296 | 1.11(0.93,1.31) 0.247 | 0.90(0.74,1.09) 0.278 |  | 0.97(0.92,1.03) 0.331 |  |

Model：Adjusted for age, sex, race, Townsend deprivation index, BMI, smoke, alcohol, cardiovascular disease, diabetes, fish, fruit, vegetable, processed meat, unprocessed meat.

**Table S11**. Joint associations of fish oil supplementation and PA with incident PD in female after excluding participants who developed PD within two years of study(n=202996)

|  | Category | | | | | Continues | |
| --- | --- | --- | --- | --- | --- | --- | --- |
|  | Q1 | Q2 | Q3 | Q4 | *P* for interaction | Per1000 Met increase | *P* for interaction |
|  | Physical activity (Sum) | | | | |  |  |
| Using fish oil supplement |  |  |  |  |  |  |  |
| Yes | Ref | 0.76(0.54,1.08) 0.124 | **0.66(0.46,0.93) 0.019** | 0.79(0.56,1.10) 0.162 | 0.3186 | 0.97(0.93,1.03) 0.313 | 0.8457 |
| No | Ref | 0.94(0.73,1.22) 0.647 | 0.98(0.76,1.26) 0.865 | 0.86(0.66,1.13) 0.282 |  | 0.98(0.95,1.02) 0.398 |  |
| Using fish oil supplement | Physical activity (Walking) | | | | |  |  |
| Yes | Ref | 1.08(0.78,1.49) 0.653 | 0.91(0.64,1.31) 0.623 | 0.82(0.55,1.23) 0.337 | 0.9048 | 0.93(0.82,1.05) 0.224 | 0.4698 |
| No | Ref | 1.04(0.82,1.32) 0.746 | 0.87(0.66,1.15) 0.335 | 0.93(0.69,1.24) 0.609 |  | 0.98(0.89,1.07) 0.627 |  |
| Using fish oil supplement | Physical activity (Moderate) | | | | |  |  |
| Yes | Ref | 0.71(0.50,1.00) 0.048 | **0.64(0.44,0.91) 0.014** | 0.76(0.54,1.05) 0.097 | 0.8030 | 0.97(0.88,1.07) 0.583 | 0.9637 |
| No | Ref | 0.88(0.68,1.13) 0.299 | **0.74(0.56,0.97) 0.032** | 0.93(0.72,1.20) 0.580 |  | 0.98(0.91,1.06) 0.562 |  |
| Using fish oil supplement | Physical activity (Vigorous) | | | | |  |  |
| Yes | Ref | 0.89(0.61,1.28) 0.524 | 0.78(0.57,1.07) 0.124 | 0.87(0.61,1.22) 0.411 | 0.4834 | 0.97(0.85,1.09) 0.591 | 0.8288 |
| No | Ref | 0.85(0.63,1.14) 0.271 | 1.00(0.80,1.27) 0.972 | 0.78(0.58,1.04) 0.091 |  | 0.96(0.87,1.06) 0.394 |  |

Model：Adjusted for age, sex, race, Townsend deprivation index, BMI, smoke, alcohol, cardiovascular disease, diabetes, fish, fruit, vegetable, processed meat, unprocessed meat.


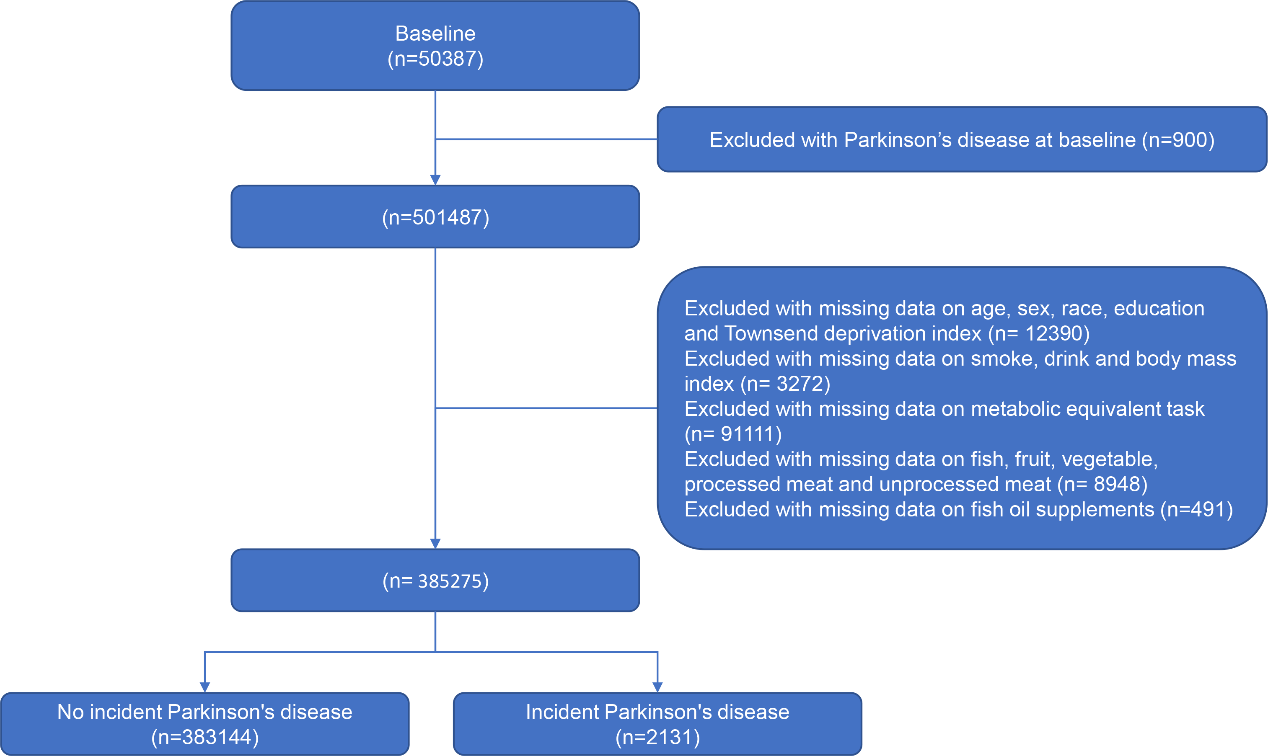


Figure S1. Flowchart of participant included.


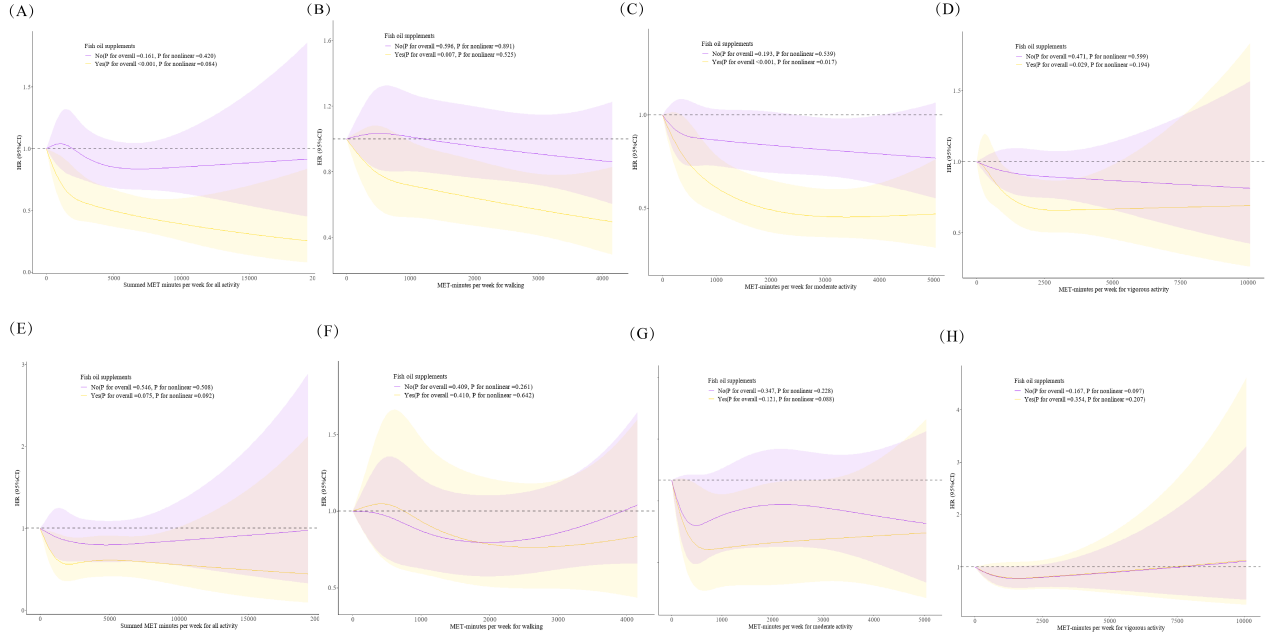


Figure S2. Dose–response associations between Summed MET minutes per week for all activity and fish oil supplement with PD incidence in males (A). Dose–response associations between MET minutes per week for walking and fish oil supplement with PD incidence in males (B). Dose–response associations between MET minutes per week for moderate activity and fish oil supplement with PD incidence in males (C). Dose–response associations between MET minutes per week for vigorous activity and fish oil supplement with PD incidence in males (D). Dose–response associations between Summed MET minutes per week for all activity and fish oil supplement with PD incidence in females (E). Dose–response associations between MET minutes per week for walking and fish oil supplement with PD incidence in females (F). Dose–response associations between MET minutes per week for moderate activity and fish oil supplement with PD incidence in females (G). Dose–response associations between MET minutes per week for vigorous activity and fish oil supplement with PD incidence in females (H). The analysis was performed after adjusting for age, race, Townsend deprivation index, BMI, smoke, alcohol, cardiovascular disease, diabetes, fish, fruit, vegetable, processed meat, unprocessed meat. 95%CI, 95% confidence interval; HR, hazard ratio; MET, metabolic equivalent task.
